# Global Metabolomic Profiling Reveals an Association of Metal Fume Exposure and Plasma Unsaturated Fatty Acids

**DOI:** 10.1371/journal.pone.0077413

**Published:** 2013-10-15

**Authors:** Yongyue Wei, Zhaoxi Wang, Chiung-yu Chang, Tianteng Fan, Li Su, Feng Chen, David C. Christiani

**Affiliations:** 1 Department of Environmental Health, Harvard School of Public Health, Harvard University, Boston, Massachusetts, United States of America; 2 Department of Epidemiology and Biostatistics, Key Laboratory of Modern Toxicology of Ministry of Education, School of Public Health, Nanjing Medical University, Nanjing, Jiangsu, China; University College London, United Kingdom

## Abstract

**Background:**

Welding-associated air pollutants negatively affect the health of exposed workers; however, their molecular mechanisms in causing disease remain largely unclear. Few studies have systematically investigated the systemic toxic effects of welding fumes on humans.

**Objectives:**

To explore the effects of welding fumes on the plasma metabolome, and to identify biomarkers for risk assessment of welding fume exposure.

**Methods:**

The two-stage, self-controlled exploratory study included 11 boilermakers from a 2011 discovery panel and 8 boilermakers from a 2012 validation panel. Plasma samples were collected pre- and post-welding fume exposure and analyzed by chromatography/mass spectrometry.

**Results:**

Eicosapentaenoic or docosapentaenoic acid metabolic changes post-welding were significantly associated with particulate (PM_2.5_) exposure (*p*<0.05). The combined analysis by linear mixed-effects model showed that exposure was associated with a statistically significant decline in metabolite change of eicosapentaenoic acid [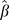
(95% CI) = −0.013(−0.022∼−0.004); *p* = 0.005], docosapentaenoic acid n_3_ [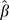
(95% CI) = −0.010(−0.018∼−0.002); *p* = 0.017], and docosapentaenoic acid n_6_ [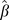
(95% CI) = −0.007(−0.013∼−0.001); *p* = 0.021]. Pathway analysis identified an association of the unsaturated fatty acid pathway with exposure (*p*_Study_−_2011_ = 0.025; *p*_Study_−_2012_ = 0.021; *p*_Combined_ = 0.009). The functional network built by these fatty acids and their interactive genes contained significant enrichment of genes associated with various diseases, including neoplasms, cardiovascular diseases, and lipid metabolism disorders.

**Conclusions:**

High-dose exposure of metal welding fumes decreases unsaturated fatty acids with an exposure-response relationship. This alteration in fatty acids is a potential biological mediator and biomarker for exposure-related health disorders.

## Introduction

The impact of welding-associated air pollutants on the health of exposed workers remains a major concern in occupational medicine and public health [1]. Exposure to fine particles (PM_2.5_) in ambient air increases the risk for acute disorders [2]–[5] and chronic diseases [6]–[10]. Ultra-fine and fine welding particles often contain various metals as a result of combustion; these include iron, nickel, sulfur, copper, vanadium, cadmium, and their oxides [11]–[13]. Metal particles may induce diverse biological effects, including activation of mitogen-activated protein kinase [14], DNA damage and cell apoptosis [5], [15], [16], lipid peroxidation [16], [17], and alteration of gene expression [18]. Further, fine metal particles inhaled into the respiratory tract translocate to blood circulation, which ultimately deposits particles in other organs and produces systemic toxic effects.

Recently, metabolomics has attracted increasing interest in the field of toxicology because it is a fast and reproducible method that directly reflects biological events [19], [20]. Metabolomics uses a non-targeted approach to obtain an accurate representation of the metabolome, including small molecules involved in highly complex biochemical networks; global changes in the metabolome may relate to particular diseases or phenotypes [21], [22]. The use of peripheral fluids, such as plasma or urine, for metabolomic analysis makes it an attractive method for studying the toxic effects of various exposures such as welding fumes. To date, however, few studies have investigated the systemic toxic effects of welding fumes on humans.

Taking advantage of our well-established occupational cohort of male boilermaker (welder) construction workers, we performed an exploratory metabolomics study using a two-stage, self-controlled design to explore the systemic toxic effects of welding fumes measured as metabolic changes. This study identified biological biomarkers for health risk assessment of welding fume exposure.

## Materials and Methods

### Ethics Statement

The Institutional Review Board at the Harvard School of Public Health approved the study protocol, and informed written consent was obtained from each adult prior to participation.

### Study Design and Population

Eleven non-smoking, non-diabetic boilermakers at an apprentice welding school (Union Local 29, Quincy, MA) were recruited in 2011 to participate in a discovery study (Study-2011); five of the same subjects from Study-2011 and three new subjects were recruited in 2012 (Study-2012) for validation. All participants were selected from the well-characterized occupational cohort of male boilermaker construction workers in Eastern Massachusetts as previously described [23]. The welding school consists of a large, temperature-controlled room outfitted with ten workstations where the welders receive instruction and perform welding, cutting, and grinding techniques. Boilermakers primarily performed shielded metal arc (stick) and gas metal arc welding (TIG), using base metals of mild steel (manganese alloys) and stainless steel (manganese, chromium, and nickel alloys) with electrodes composed mainly of iron with variable amounts of manganese (1∼5%). The same breakfast and lunch including turkey, chicken, ham sandwiches and vegetables was provided for each participant during workshop to control the inter-subject variability.

### Data Collection

Peripheral blood samples were collected before (pre) and immediately after the ∼5 h welding workshop (post) from all subjects. Samples were inventoried and immediately stored at −80°C. Samples of personal, integrated, gravimetric particulate matters with an aerodynamic diameter of ≤2.5 mm (PM_2.5_) were collected over the duration of the welding workshop. A self-administered questionnaire collected information on medical history, medication use, demographics, and smoking history as previously described [13].

### Metabolite Profiling

#### Sample preparation

Frozen samples were sent to Metabolon, Inc. (Durham, NC) and accessioned into the Metabolon LIMS system by a unique identifier associated with the original source only. Samples were prepared using the automated MicroLab STAR® system (Hamilton Company, Reno, NV). Recovery standards were added prior to the first step in the extraction process for quality control purposes. Sample preparation used a proprietary series of organic and aqueous extractions to remove proteins while allowing maximum recovery of small molecules. The resulting extract was divided into two fractions: one for analysis by liquid chromatography (LC), and one for analysis by gas chromatography (GC). Samples were placed briefly on a TurboVap® (Zymark, Westborough, MA) to remove organic solvents, frozen, and dried under vacuum. Extracted samples were split into equal parts for analysis by gas chromatography/mass spectrometry (GC/MS) and liquid chromatography/mass spectrometry (LC/MS) platforms. Several technical replicate samples were created from a homogeneous pool containing a small amount of each sample.

#### Instrument variability control

Instrument variability was determined by calculating the median relative standard deviation (RSD) for internal standards added to each sample prior to mass spectrometer injection (RSD = 5%). Overall process variability was determined by calculating the median RSD for all endogenous metabolites (i.e., non-instrument standards) present in pooled technical replicates (RSD = 14%). Both RSD values meet Metabolon’s acceptance criteria.

#### Data extraction and quality assurance

Raw MS data files were loaded into a relational database. Peaks were identified using Metabolon’s proprietary peak integration software, and component parts were stored in a separate and specifically designed complex data structure.

#### Compound identification

Compounds were identified by comparison to library entries of purified standards or recurrent unknown entities. Identification of known chemical entities was based on comparison to the over 1,000 commercially available, purified standard compounds registered in LIMS for distribution to both LC and GC platforms.

#### Normalization

For studies spanning multiple days, data normalization was performed to correct variation resulting from instrument inter-day tuning differences. Each compound was corrected in run-day blocks by equalizing the medians to 1.00 and normalizing each data point proportionately. Normalized metabolites were used for analysis.

### Statistical Analysis

Demographics were presented by frequencies for categorical variables and means ± standard deviation (mean ± SD) for continuous variables. The change of each metabolite during welding day (Δ = post-pre) was calculated for each subject. The permutation test with 10,000 perturbations was used to test global metabolic changes between post- and pre-welding workshop samples.

To investigate the association between metabolic change and PM_2.5_ exposure, univariate linear regression was used in Study-2011 (Eq. 1). Compounds with *p*≤0.05 were further validated in Study-2012 using the same statistical model, followed by combined analysis of both studies using linear mixed-effects model (LMM) given a random slope of exposure for each study and autoregressive correlation structure (Eq. 2). The multiple comparison was adjusted by false discovery rate (FDR) [24]

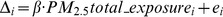
(1)where *i* = 1, …, 11 in Study-2011 and *i* = 1, …, 8 in Study-2012.
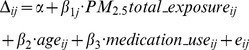
(2)where 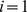, …, 14 to represent each subject; 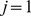
or 2 to denote each study (1 = Study-2011; 2 = Study-2012).

For pathway-level association analysis, principal component analysis (PCA) was performed to generate the first principal component (PC_1_) from all metabolites of each pathway. PC_1_ was used as the response to perform linear regression with exposure in Study-2011 and Study-2012 separately, and perform LMM for combined analysis with adjustment for age and medication use. LMM analysis was performed by R “*nlme”* package. All statistical analyses were performed using R Version 15.0 (http://cran.r-project.org).

The functional network was built using MetaCore™ online software (GeneGo Inc., Carlsbad, CA) to illustrate potential biological connections of significant compounds and interactive genes. Enrichment analysis for disease-associated genes was conducted for each network in MetaCore™. The *p* value for enrichment analysis was calculated using Eq. 3:
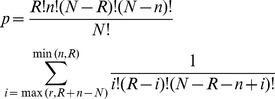
(3)where *N* denotes the total number of genes that were causally associated with all diseases and disorders in MetaCore™; *n* denotes the total number of genes that were causally associated with the intersecting disease of interest (e.g., lipid metabolism disorders); *R* denotes the total number of genes/proteins visible in the network; and *r* denotes the total number of genes/proteins visible in the network intersecting with the disease of interest.

## Results

Study-2011 consisted of eleven male boilermakers with a mean age of 49.1±10.2 years, average body mass index (BMI) of 26.5±2.9, and of whom 82% were white (Table 1). Three participants reported medical history, two with hyperlipidemia and one with hypertension; four participants reported medication use for high cholesterol, seasonal allergy, asthma, etc. Eight boilermakers were recruited in Study-2012 for validation; the characteristics of this cohort did not significantly differ from Study-2011 (Table 1). No participants reported metal fever during welding workshop.

**Table 1:** Characteristics of the study population.^a^

Characteristic		Study-2011 (*n* = 11)	Study-2012 (*n* = 8)^b^
PM_2.5_ total exposure		74.2±27.4	114.9±59.7
Age (years)^c^		49.1±10.2	46.1±11.7
BMI (kg/m^2^)		26.5±2.9	28.1±3.7
Male		11	8
Race	*White*	9	6
	*Black*	1	0
	*Hispanic*	1	2
Medical history		3	4
	*Diabetes*	0	0
	*Asthma*	0	1
	*High blood pressure*	1	1
	*High cholesterol hyperlipidemia*	2	2
Medication use		4	4
	*Simvastatin for high cholesterol*	2	1
	*Omeprazole for acid reflux*	1	1
	*Lisinopril for high blood pressure*	0	1
	*Fexofenadine for allergy*	1	1
	*Ibuprofen for headaches*	1	0
	*Albuterol for asthma*	0	1
	*Oxycodone for fractured back*	0	1

a Values presented either as mean±SD or *n*;

b Five subjects participated in both studies;

c At study entry.

### Single Biochemical Analysis

**Figure 1:**
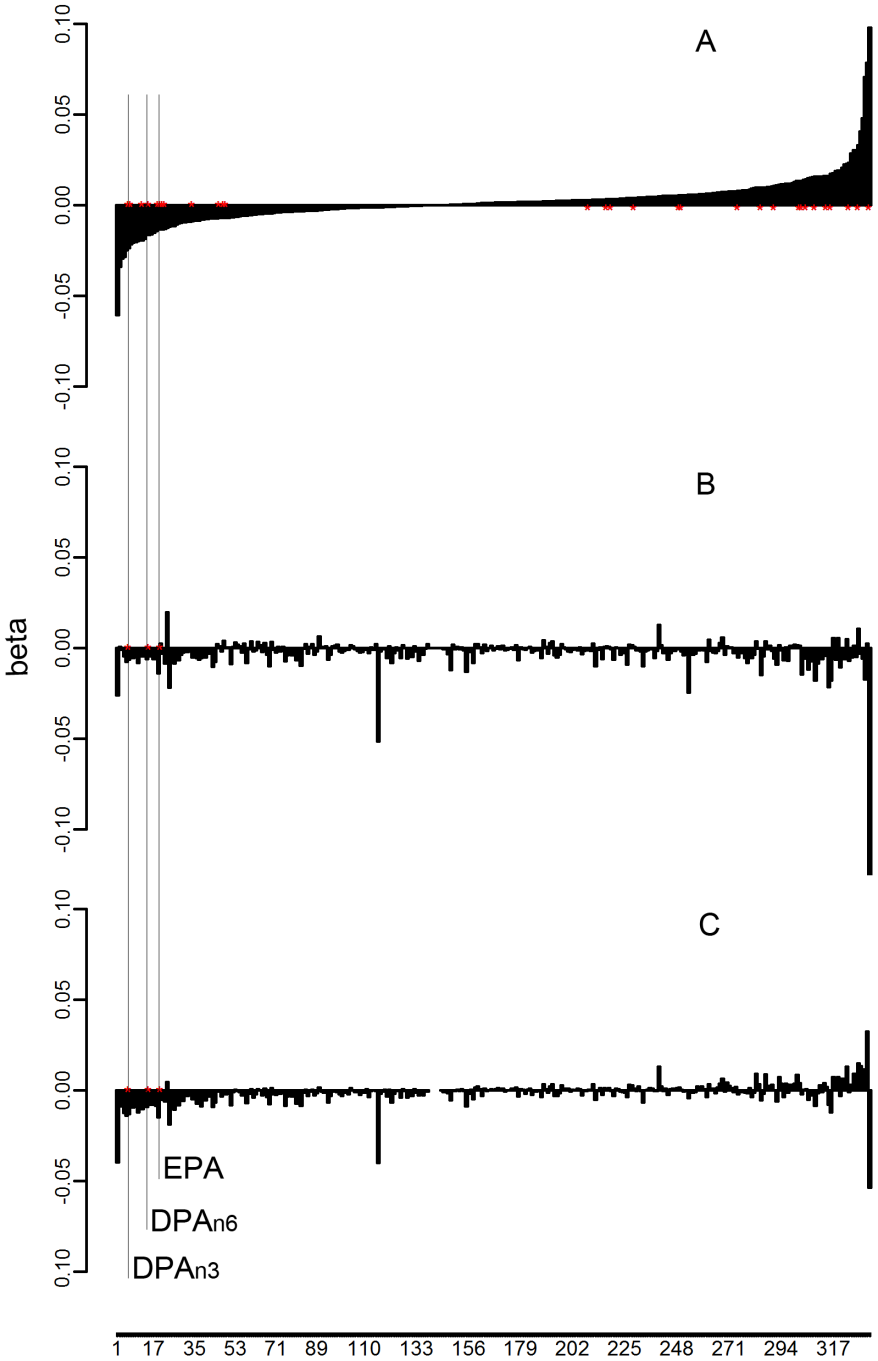
Flowchart for single-compound analysis. Metabolite changes (post-welding workshop – pre-welding workshop) for 333 compounds were analyzed by regression with PM_2.5_ total exposure in Study-2011 (A) and Study-2012 (B), and by linear mixed-effects model in the combined dataset of both studies (C). The *y*-axis represents the coefficients of exposure in regression models. The *x*-axis represents the metabolites ordered by the effects of exposure from discovery analysis. Thirty biochemical metabolic changes significantly associated with exposure (*p*<0.05) in Study-2011. Three of the thirty changes were validated in Study-2012 (*p*<0.05) and remained significant in combined analysis: eicosapentaenoic acid (EPA) and docosapentaenoic acids (DPA_n3_, DPA_n6_).

**Figure 2:**
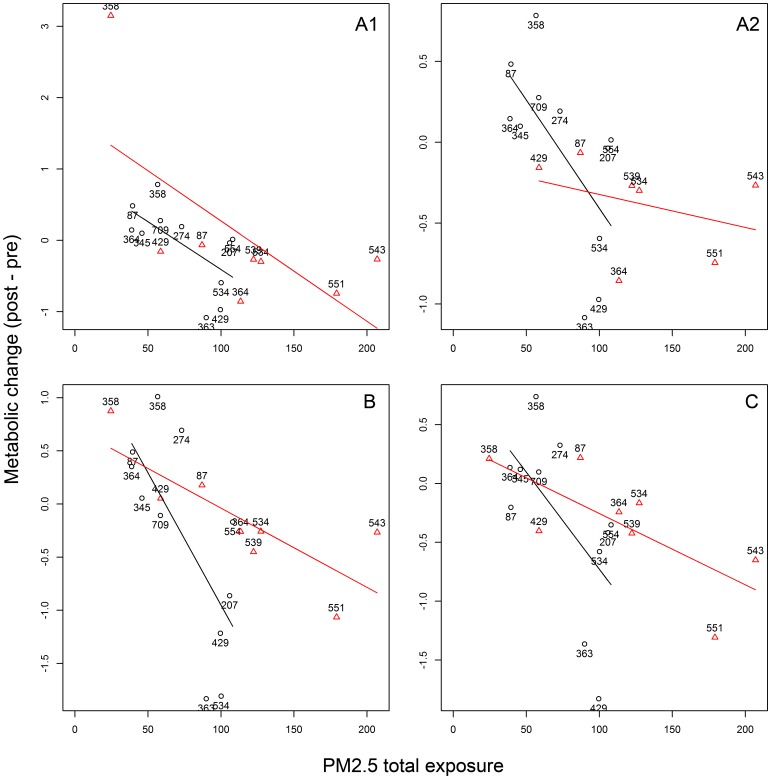
Scatter plots of metabolic changes by exposure of EPA and DPA. Scatter plots illustrate the biochemical compounds that had a metabolic change significantly associated with welding fume exposure: A1) eicosapentaenoic acid (EPA); A2) EPA after removal of a potential outlier (subject ID: 358); B) docosapentaenoic acid n_3_ (DPA_n3_); and C) docosapentaenoic acid n_6_ (DPA_n6_). The *x*-axis represents total PM_2.5_ exposure during the welding workshop, while the *y*-axis represents biochemical metabolic change (post-welding workshop – pre-welding workshop). Black circles represent data from Study-2011; red triangles represent data from Study-2012. Each mark is labeled with the subject ID.

Plasma samples were collected from each participant prior to and after a welding workshop for metabolomic profiling. We identified 333 compounds of known biochemical identity that comprised the analysis dataset. In Study-2011, 30 of 333 detected compounds were significantly associated with a metabolic change during welding workshop (Δ) with total PM_2.5_ exposure (*p*≤0.05); however, only three passed validation in Study-2012 by the criteria of consistent effect direction of exposure and *p*≤0.05 (Figure 1, Table 2). The combined LMM analysis showed that total PM_2.5_ exposure associated with a statistically significant (*p*<0.05) decline in metabolic change of eicosapentaenoic acid (EPA) (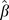 = −0.013/ µg/m^3^; 95% CI = −0.022∼−0.004; *p* = 0.005; *q* = 0.249), docosapentaenoic acid n_3_ (DPA_n3_) (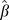 = −0.010/ µg/m^3^; 95% CI = −0.018∼−0.002; *p* = 0.017; *q* = 0.313), and docosapentaenoic acid n_6_ (DPA_n6_) (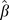 = −0.007/ µg/m^3^; 95% CI = −0.013∼−0.001; *p* = 0.021; *q* = 0.313) (Figure 2). Adjustment for age and medication use did not affect significance (Table 3). LMM application with random intercepts for each study provided consistent results; however, the model did not reach convergence when random intercept and random slope were simultaneously considered.

**Table 2:** Metabolites associated with PM_2.5_ metal welding fume exposure.

	Baseline		Δ (post - pre)	
Metabolite	Study-2011	Study-2012	Study-2011	Study-2012
Eicosapentaenoate (EPA; 20∶5n3)	1.06±0.44(0.45∼1.89)	1.42±0.67(0.70∼2.88)	−0.06±0.58(−1.08∼0.78)	0.06±1.28(−0.86∼3.15)
Docosapentaenoate (DPA_n3_; 22∶5n3)	1.29±0.71(0.45∼2.68)	1.09±0.29(0.65∼1.48)	−0.31±0.98(−1.83∼1.01)	−0.15±0.56(−1.07∼0.87)
Docosapentaenoate (DPA_n6_; 22∶5n6)	1.32±0.60(0.52∼2.4)	1.10±0.33(0.70∼1.67)	−0.30±0.75(−1.83∼0.74)	−0.35±0.49(−1.31∼0.22)

* Values presented as mean ± SD (min∼max).

**Table 3:** Association of PM_2.5_ metal welding fume exposure with metabolite change.

	Association analysis a				Combined analysis a,b					
	Study-2011		Study-2012		Univariate analysis			Multivariate analysis		
Metabolite	*β*(95% CI)	*p*	*β*(95% CI)	*p*	*β*(95% CI)	*p*	*q* c	*β*(95% CI)	*p*	*q* c
Eicosapentaenoate (EPA; 20:5n3)	−0.013(−0.026,−0.001)	0.038	−0.014(−0.027,−0.001)	0.033	−0.013(−0.022,−0.004)	0.005	0.249	−0.014(−0.023,−0.006)	0.003	0.229
Docosapentaenoate (DPA_n3_; 22:5n3)	−0.025(−0.044,−0.005)	0.018	−0.007(−0.013,−0.002)	0.017	−0.010(−0.018,−0.002)	0.017	0.313	−0.010(−0.019,−0.001)	0.020	0.398
Docosapentaenoate (DPA_n6_; 22:5n6)	−0.016(−0.032,−0.0002)	0.048	−0.006(−0.012,−0.0005)	0.038	−0.007(−0.013,−0.001)	0.021	0.313	−0.021(−0.013,−0.001)	0.029	0.418

a Total PM_2.5_ exposure as predictor, metabolite change as response;

b Linear mixed-effects model was used with/without adjustment for age and medication use;

c *q* represented FDR adjusted *p* value using Benjamini & Hochberg method.

In Study-2012, one subject (ID: 358) exhibited EPA metabolic changes much larger than others. These data were therefore removed for subsequent sensitivity analysis, which displayed a consistent trend while losing statistical significance (*p* = 0.447); the combined analysis remained statistically significant in univariate analysis (*p* = 0.030) and multivariate analysis (*p* = 0.041) (Figure 2-A2).

The five subjects who participated in both studies were also separately assessed by sensitivity analysis using an LMM with random slopes for each subject. Borderline statistical significance remained for EPA (*p*_Study-2011_ = 0.070; *p*_Study-2012_ = 0.088; *p*_Combined_ = 0.042; *p*_Combined_ = 0.012 with adjustment for age and medication use) and DPA_n3_ (*p*_Study-2011_ = 0.041; *p*_Study-2012_ = 0.031; *p*_Combined_ = 0.003; *p*_Combined_ = 0.066 with adjustment for age and medication use). LMM with random intercepts for each subject yielded similar results.

Conversely, sensitivity analysis was performed excluding the five subjects from Study-2011 who participated in both studies; this analysis included six subjects for Study-2011 and eight subjects for Study-2012. Combined analysis remained significant or borderline significant for EPA (*p* = 0.046), DPA_n3_ (*p* = 0.064), and DPA_n6_ (*p* = 0.020), even after adjustment for age and medication use.

### Pathway Analysis

EPA, DPA_n3_, and DPA_n6_ participate in the biosynthesis of unsaturated fatty acids (KEGG entry: map01040). To explore whether this pathway connects metabolites associated with PM_2.5_ metal fume exposure, we performed PCA to integrate multiple biochemicals within each pathway. While two of 63 pathways significantly associated with PM exposure (*p*≤0.05) in Study-2011, only the unsaturated fatty acids pathway was replicated in Study-2012 (*p*_Study-2011_ = 0.025; *p*_Study-2012_ = 0.021) and appeared in the combined analysis (*p* = 0.009, *q* = 0.145; *p*_adj_ = 0.013, *q* = 0.204) (Table 4). Seven biochemical molecules were detected in the unsaturated fatty acids pathway for which metabolic change negatively associated with exposure dosage; six of the seven molecules were statistically significant by combined analysis of both studies (Table S1).

**Table 4:** Association of PM_2.5_ metal welding fume exposure with metabolite pathways.

				Study-2011^d^		Study-2012^d^		Combined analysis^f^		
Pathway	*N* a	*N*_2_ b	*N*_3_ c	*VPC*_1_ e	*p*	*VPC*_1_ e	*p*	*VPC*_1_ e	*p*	*p*_adj_
Unsaturated fatty acid	7	7	6	0.84	0.025	0.81	0.021	0.77	0.009	0.013
Phenylalanine & tyrosine metabolism	13	8	1	0.29	0.013	0.36	0.547	0.24	0.425	0.480

a Number of biochemical compounds within the same pathway;

b Number of biochemical compounds that have the same coefficient direction when regressed by exposure both in Study-2011 and Study-2012;

c Number of biochemical compounds with association *p<*0.05 in combined dataset analyzed by linear mixed model with adjustment of age and medication use;

d First principal component (PC_1_) was used as response, with PM_2.5_ total exposure as predictor in linear regression model or linear mixed-effects model;

e Proportion of variance explained by PC_1_;

f PC_1_ was used as response in the linear mixed model with random slope with or without adjustment of age and medication use.

### Functional Network Analysis

**Figure 3:**
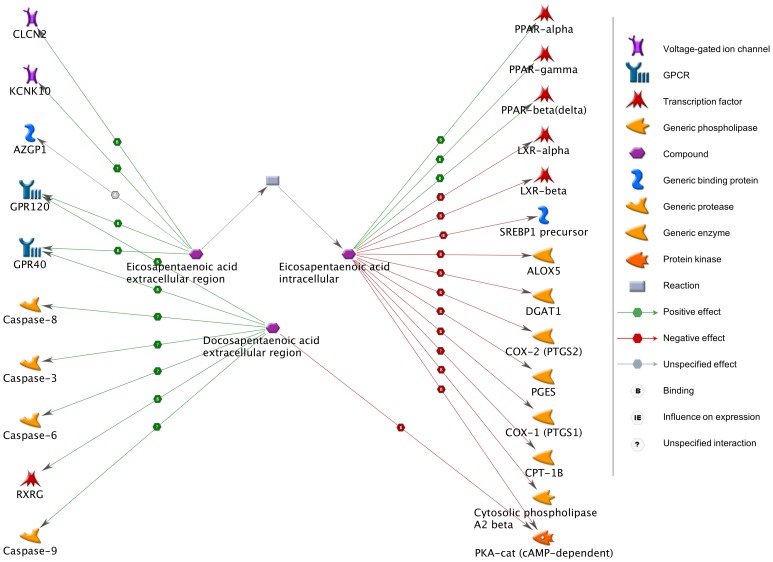
Functional network for EPA and DPA. Network analysis revealed that intracellular and extracellular eicosapentaenoic acid (EPA) interact with 19 genes, while docosapentaenoic acid (DPA) interacts with 7 genes; EPA, DPA_n3_, DPA_n6_, and 24 regulated genes were used to build the illustrated functional network. The green line represents activation; the red line represents inhibition; the gray line represents unspecified effects.

To explore interactions of EPA and DPA with specific genes, we performed a functional network analysis. EPA extracellular and intracellular metabolites interacted with 19 genes; DPA interacted with 7 genes. All metabolite compounds and 24 interactive genes were then used to build a network (Figure 3). Using *a priori* knowledge, the top five diseases that associated with the most interactive genes within the network (21/24 genes) were endocrine system diseases (*p* = 1.6×10^−60^), neoplasms (*p* = 2.0×10^−59^), lung disease (*p* = 2.0×10^−59^), respiratory tract diseases (*p* = 2.0×10^−59^), and digestive system diseases (*p* = 2.0×10^−59^). Additionally, 16 of 24 genes associated with cardiovascular disease (*p* = 1.3×10^−41^), and 9 of 24 genes associated with lipid metabolism disorders/hyperlipidemias (*p* = 2.2×10^−27^). These disease enrichment profiles remained significant when all seven compounds were used to build a network by the same approach.

## Discussion

A challenge to understand the adverse effects of welding fume exposure is the complex nature of environmental and behavioral influences. Metabolic profiles are greatly affected by diet, smoking behavior, medical history, or even time [25]–[29]. Kuo *et al.* recruited non-smoking, non-alcohol drinking welders for a metabolomic study, but intra-subject variation was not considered [30]. Here, we excluded smoking or diabetic boilermakers to reduce such confounding effects. A self-controlled design eliminated intra-subject variation, and a two-stage design avoided chance findings in our limited sample size.

The main finding of this study is that unsaturated fatty acids are consistently reduced by respirable welding fume exposure, with a significant negative exposure-response relationship. To date, many studies have investigated the beneficial effects of EPA and DHA in neural function [31], tumor suppression [32], cardiovascular event risk reduction [33], diabetes mellitus [34], [35], anti-inflammatory activity [36], and blood lipid reduction [37]. Although the roles of DPA have not yet been systematically examined because of the lack of available pure DPA, there is some evidence that DPA has stronger beneficial health effects than EPA and DHA *in vitro* [38]. We also detected a global metabolite change, which may be explained by diet during work shift, circadian variation [30], or PM_2.5_ exposure.

Surprisingly, the functional network built from unsaturated fatty acids includes 24 genes that showed very intense biological functions related to various diseases, including those involving endocrine system diseases, respiratory diseases, neoplasms, cardiovascular diseases, and lipid metabolism disorders. Intracellular EPA activates peroxisome proliferator-activated receptors (PPARs) [39], which are implicated in the pathology of numerous diseases including obesity, diabetes, atherosclerosis, and cancer (RefSeq, Jul 2008). EPA also inhibits *COX-1/COX-2* [40], which encode proteins that may promote cell proliferation during tumor progression (RefSeq, Sep 2012). DPA activates *RXRG* [41], a member of the retinoid X receptor family that is involved in mediating the anti-proliferative effects of retinoic acid and is down-regulated in several types of human cancers, including lung cancer [42]; DPA also activates cysteine-aspartic acid protease (Caspase) family members, which are involved in cancer cell apoptosis [43]. The functional network analysis strengthens the hypothesis that the decline of unsaturated fatty acids is a potential mediator of multiple health disorders in boilermakers.

Previous studies demonstrate the association of welding fume exposure on decline of heart rate variability (HRV), an important indicator of cardiovascular disease morbidity [4], [44], [45]. Interestingly, several recent studies show that supplementation of EPA and DPA benefit HRV in healthy adults, cardiovascular disease risk populations, or in cardiovascular disease patients [46]–[50]. These results strongly indicate that the metabolic change of unsaturated fatty acids is an important biological mediator of exposure-related decreases in HRV.

Despite these findings, we recognize limitations to the study: we lack metabolite information from a non-welding day, and *p* values were not small enough to pass multiple comparison correction due to small sample size. However, pathway-based analysis showed an acceptable FDR (*q*<0.2), and the two-stage, self-controlled study design helped reduce chance findings.

In summary, this exploratory study shows evidence that high dose exposure of metal welding fumes decreases unsaturated fatty acids with an exposure-response relationship. The metabolic change in unsaturated fatty acids is a potential biomarker for exposure-related health disorders for which further studies are encouraged.

## Supporting Information

Table S1 Association analysis for metal welding fume exposure and metabolic change of biochemical compounds of unsaturated fatty acid pathway.
(DOCX)
