# Supplementary material for: Global Metabolomic Profiling Reveals an Association of Metal Fume Exposure and Plasma Unsaturated Fatty Acids
Source: PLoS One. 2013 Oct 15;8(10):e77413. doi: 10.1371/journal.pone.0077413 (PMC3797131; doi:10.1371/journal.pone.0077413)
Supplement: Table S1 — Association analysis for metal welding fume exposure and metabolic change of biochemical compounds of unsaturated fatty acid pathway. (DOCX) [file pone.0077413.s001.docx]

Table S1. Association analysis for metal welding fume exposure and metabolic change of biochemical compounds of unsaturated fatty acid pathway.

|  | Baseline ^a^ | | Change ^a^ | | Association analysis ^b^ | | | | Combined analysis ^b, c^ | | | |
| --- | --- | --- | --- | --- | --- | --- | --- | --- | --- | --- | --- | --- |
|  |  |  |  |  | Study-2011 | | Study-2012 | | Univariate model | | Multivariate model | |
| Metabolite | Study-2011 | Study-2012 | Study-2011 | Study-2012 | *β*(95% *CI*) | *p* | *β*(95% *CI*) | *p* | *β*(95% *CI*) | *p* | *β*(95% *CI*) | *p* |
| Eicosapentaenoate (EPA; 20:5n3) | 1.06±0.44(0.45~1.89) | 1.42±0.67(0.7~2.88) | -0.06±0.58(-1.08~0.78) | 0.06±1.28(-0.86~3.15) | -0.013(-0.026,-0.001) | 0.038 | -0.013(-0.030,-0.002) | 0.005 | -0.013(-0.021,-0.005) | 0.004 | -0.014(-0.022,-0.006) | 0.003 |
| Docosapentaenoate (DPA_n3_; 22:5n3) | 1.29±0.71(0.45~2.68) | 1.09±0.29(0.65~1.48) | -0.31±0.98(-1.83~1.01) | -0.15±0.56(-1.07~0.87) | -0.025(-0.044,-0.005) | 0.018 | -0.025(-0.013,-0.002) | 0.017 | -0.010(-0.018,-0.003) | 0.017 | -0.01(-0.018,-0.003) | 0.020 |
| Docosapentaenoate (DPA_n6_; 22:5n6) | 1.32±0.6(0.52~2.4) | 1.1±0.33(0.7~1.67) | -0.3±0.75(-1.83~0.74) | -0.35±0.49(-1.31~0.22) | -0.016(-0.033,-0.0002) | 0.048 | -0.016(-0.012,-0.001) | 0.021 | -0.007(-0.013,-0.002) | 0.019 | -0.007(-0.013,-0.001) | 0.029 |
| Dihomo-gamma-linolenic acid (20:3n3 or n6) | 1.22±0.46(0.57~1.84) | 1.13±0.60(0.66~2.36) | -0.16±0.60(-1.31~0.51) | -0.33±0.60(-1.71~0.18) | -0.014(-0.027,-0.002) | 0.030 | -0.014(-0.014,-0.003) | 0.015 | -0.007(-0.012,-0.002) | 0.015 | -0.007(-0.012,-0.002) | 0.022 |
| Docosahexaenoate (DHA; 22:6n3) | 1.29±0.72(0.3~3.11) | 1.23±0.44(0.69~2.05) | -0.23±0.93(-2.44~0.93) | -0.16±0.65(-0.92~1.23) | -0.020(-0.041,-0.001) | 0.062 | -0.02(-0.0315-0.0009) | 0.019 | -0.010(-0.017,-0.002) | 0.019 | -0.010(-0.018,-0.002) | 0.028 |
| Linoleic acid (18:2n6) | 1.04±0.49(0.34~2.06) | 0.81±0.34(0.4~1.4) | 0.2±0.74(-0.97~1.75) | 0.13±0.46(-0.78~0.86) | -0.015(-0.032,-0.002) | 0.070 | -0.015(-0.011,-0.001) | 0.022 | -0.007(-0.013,-0.002) | 0.022 | -0.007(-0.013,-0.002) | 0.020 |
| Linolenic acid (α or γ; 18:3n3 or 6) | 1.05±0.61(0.18~1.92) | 0.71±0.39(0.29~1.24) | 0.22±1.08(-1.51~2.55) | 0.25±0.45 (-0.52~0.91) | -0.021(-0.046,0.003) | 0.081 | -0.021(-0.011,0.0003) | 0.087 | -0.008(-0.016,0.001) | 0.087 | -0.008(-0.016,0.0001) | 0.067 |

^a^ Values presented as mean± SD (min~max) for normalized metabolite level;

^b^ Total PM_2.5_ exposure as predictor, metabolite level as response;

^c^ Linear mixed-effects model with a random slope and autoregressive correlation structure was used with/without adjustment of age and medication use.
